# Precaution, governance and the failure of medical implants: the ASR^(TM)^ hip in the UK

**DOI:** 10.1186/s40504-014-0019-2

**Published:** 2014-11-26

**Authors:** Matthias Wienroth, Pauline McCormack, Thomas J Joyce

**Affiliations:** Centre for Forensic Science, University of Northumbria at Newcastle, Northumberland Building, Northumberland Road, Newcastle upon Tyne, NE1 8ST UK; Policy, Ethics & Life Sciences Research Centre, Newcastle University, Claremont Bridge, Claremont Road, Newcastle upon Tyne, NE1 7RU UK; School of Mechanical and Systems Engineering, Newcastle University, Stephenson Building, Claremont Road, Newcastle upon Tyne, NE1 7RU UK

**Keywords:** Medical implant failure, Medical device, Governance, Precautionary principle, DePuy ASR, Metal-on-metal hips

## Abstract

Hip implants have provided life-changing treatment, reducing pain and improving the mobility and independence of patients. Success has encouraged manufacturers to innovate and amend designs, engendering patient hopes in these devices. However, failures of medical implants do occur. The failure rate of the Articular Surface Replacement metal-on-metal hip system, implanted almost 100,000 times world-wide, has re-opened debate about appropriate and timely implant governance. As commercial interests, patient hopes, and devices’ governance converge in a socio-technical crisis, we analyse the responses of relevant governance stakeholders in the United Kingdom between 2007 and 2014. We argue that there has been a systemic failure of the governance system entrusted with the safety of patients fitted with medical implants. Commercial considerations of medical implants and the status quo of medical implant governance have been given priority over patient safety despite the availability of significant failure data in an example of uncertainty about what constitutes appropriate precautionary action.

## Introduction

This paper examines a history of managing medical implant failure, specifically the responses of key stakeholders in an emergent realisation of failure. By ‘failure’ we refer to guidelines by NICE (National Institute for Health and Care Excellence) (2002) which stipulate a benchmark for hip implant revision of 10% at ten years, commonly translated to 1% per year of the life of the implant^a^. We draw on the example of DePuy Orthopaedics’ Articular Surface Replacement (ASR™) hip system to show systemic failure of medical implant governance in the United Kingdom (UK). The ASR™ is a metal-on-metal (MoM) hip implant that was withdrawn from the market by the manufacturer on 26 August 2010. High revision rates of the ASR™ – at 7.5% in three years (NJR 2009), more than twice the NICE benchmark – have led to the need to explant and replace the artificial hip due to a risk of tissue damage caused by increased wear releasing high concentrations of metal particles into the patient (Langton et al. 2011).

### Total hip replacement

**Figure 1:**
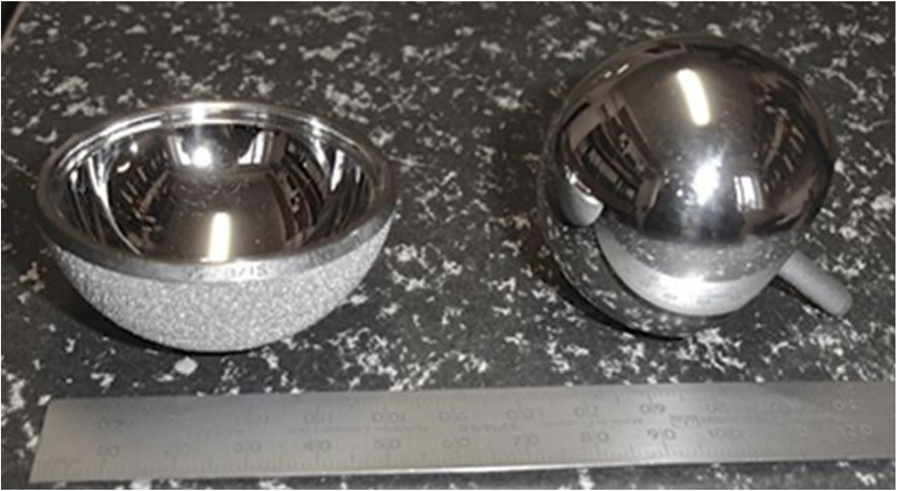
**DePuy ASR** **™** **resurfacing hip (note the small femoral stem right).** To the left is the acetabular cup and to the right is the single-piece femoral component.

Medical implants have had considerable impact on healthcare planning and provision – both on the lives of patients and in commercial relevance to manufacturers, prompting one commentator to conclude that ‘medical devices matter’ (Altenstetter 2003: 228). Total hip replacement had until very recently been celebrated as ‘the operation of the century’ (Learmonth et al. 2007: 1508) and considered to have been ‘one of the most successful operations of the 20th century’ (Skinner and Kay 2011: 3009). For the majority of recipients it is a positive, life changing intervention, leading to increased mobility and a reduction in pain. The overall success of hip joint replacement has increased trust in the process of replacement surgery and in the prostheses, leading to an exploration of unmet need in some groups of patients, which in turn has driven a desire to innovate on the part of the manufacturers. One area which was seen as promising for development was the provision of prostheses for ‘young and active patients’ (Daniel et al. 2004: 177) who are ‘likely to outlive a conventional primary total hip replacement’ (NICE 2002: 3). The challenge has been to develop artificial joints which can withstand higher levels of activity over a greater number of years than was the case previously. MoM hip designs were first tried unsuccessfully in the 1950s and 1960s until improvements in manufacturing and measurement technologies encouraged designers to revisit MoM hips in the 1990s. Around the same time, manufacturers were experimenting with a radically different design – resurfacing – where the femoral stem (Figure 1) is much smaller, resulting in greater femoral bone conservation. One of the first successful MoM hip resurfacing designs was the Birmingham Hip Resurfacing (BHR™) which showed great promise with revision rates of 0.02% (1 out of 440 implants) over a mean 3.3 years follow-up (Daniel et al. 2004).

Whilst total hip replacement has had considerable positive outcomes for patients, in some cases medical implants have caused unexpected results detrimental to their desired effect. Such adverse events raised awareness of inadequacies not only of device development and testing, but also of device governance (Freemantle 2011; Riordan et al. 1998; Sedrakyan 2012). In addition, it has been argued that new and innovative designs of hip and knee implants show no benefits over older designs (Nieuwenhuijse et al. 2014). Implants require particular care as they act in vivo and can have considerable impact on the surrounding tissue and wider metabolism. In considering shortfalls in the testing, use and governance of MoM hip implants we are reminded of similar issues which have arisen over the past two decades (cf. Anderson et al. 2007; Faulkner 2009) including: the 3M™ Capital™ hip (Muirhead-Allwood 1998; The Royal College of Surgeons of England 2001) and the Sulzer Hip system failures (Stephens et al. 2006). Despite these significant events the modes of governance of medical implants – professional, notifying and regulatory – were again shown to be lacking by the recent global failures of the DePuy ASR™ hip, while problems with MoM hips in general continue to be noted (Heneghan et al. 2012).

### Aims of this paper

There has been much discussion elsewhere of mechanisms and regulation which permit medical implants to enter the market without the need for simulator testing or clinical trials (Cohen 2011; Curfman and Redberg 2011; Heneghan et al. 2011). This paper attends to a later point in the process, and explores a question frequently articulated by patients dealing with the consequences of failed MoM hip implants (House of Commons Science and Technology Committee 2012): when data emerges indicating medical implant failure at an unacceptable level, who then is responsible for responding to those data, particularly for preventing harm to patients? By exploring the actions and rationales of key stakeholders we argue that there has been systemic failure of implant governance in the UK.

## Success to failure: An introductory history of the ASR™ case

**Figure 2:**
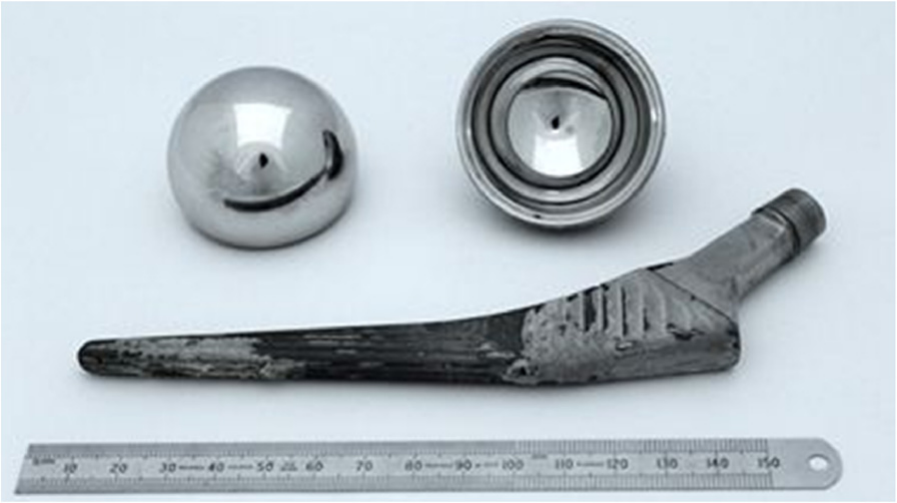
**DePuy ASR** **™** **XL total hip replacement with conventional femoral stem (shown above ruler).** Femoral head is shown top left and the acetabular cup is shown top right.

In 2003, the medical implant manufacturer DePuy Orthopaedics – a subsidiary of Johnson & Johnson – introduced their own version of a MoM hip resurfacing, ASR™ (Figure 1). DePuy also offered the ASR™ XL, a total hip replacement version (Figure 2). Both versions became available in Europe under the clause of ‘substantial equivalence’, which permits fast-track market accreditation by claiming the implant in question is similar to one already on the market (cf. The Council of the European Communities 1990: 4). The ASR™ hip resurfacing was not granted market approval by the Food and Drug Administration (FDA) in the USA. The ASR™ XL was available worldwide including the USA. It was to be these two versions that would fail in high numbers of patients causing widespread health problems (Cohen 2011).

Problems with the ASR™ in the UK were first raised at orthopaedic conferences and in journal publications in 2008. In 2009, the annual report published by the National Joint Registry of England and Wales (NJR) showed relatively high revision rates for the ASR™ hip resurfacing – 7.5% at three years (NJR 2009). However, official notices about problems with the DePuy ASR™ were limited to guidance on positioning during surgery (MHRA [Medicines and Healthcare products Regulatory Agency] 2010a) until August 2010 when DePuy Orthopaedics issued a global recall of all ASR™ XL and ASR™ hip systems (DePuy Orthopaedics Inc. 2010, MHRA 2010d).

The Medicines and Healthcare products Regulatory Agency (MHRA) had also issued a general Medical Device Alert (MDA) on 22 April 2010 for ‘All metal-on-metal (MoM) hip replacements’ (MDA/2010/033), which noted that ‘a small number of patients implanted with these hips … develop progressive soft tissue reactions to the wear debris associated with MoM articulations’ (MHRA 2010c). This MDA did not specifically address ASR™ implants but showed that data had been available indicating general concerns about MoM hip implants. It is worth noting that problems were picked up earlier in Australia, where the registry first highlighted high revision rates of the ASR™ in 2007 (Australian Orthopaedic Association, 2007) and the ASR™ was withdrawn at the end of 2009 (Australian Senate Community Affairs References Committee, 2011; Therapeutic Goods Administration, 2011). These dates are summarised in Table 1.

**Table 1:** **Simplified History of the ASR** ^**TM**^

2003	ASR™ resurfacing introduced
2007	High revision rates reported by Australian Orthopaedic Association, National Joint Replacement Registry
2008	Subsequent reporting of high revision rates by Australian Orthopaedic Association, National Joint Replacement Registry
2009	First report of high revision rates in National Joint Registry of England and Wales
	ASR™ withdrawn in Australia and New Zealand
2010	MHRA issues MDA on all MoM implants noting a small number of patients have adverse reactions
	DePuy release guidance on positioning
	MHRA issue MDA on positioning
	DePuy withdraw the ASR™ globally

MHRA Medicines and Healthcare products Regulatory Agency 2010a MHRA 2010a

The impact on patients is significant. Approximately 10,000 ASR™ hips were implanted in the UK. A failed MoM hip implant can cause both localised and systemic health problems, and symptoms such as hearing loss, dizziness, decline in cognitive function, cardiomyopathy and organ failure have been reported (Tower 2010; Mao et al. 2011). For patients, these iatrogenic effects meant increased pain and decreased mobility, affecting their social and family lives, their ability to work, to undertake hobbies and leisure activities; and often had a negative impact on their self-esteem and mental health (McCormack and Joyce 2013).

### Stakeholder responses

There are a number of organisations who play a significant role in the UK system of post-market medical implant surveillance and regulation including: the manufacturers; the main medical regulatory authority in the UK, the MHRA; the NJR database of implanted hip and knee joints; and orthopaedic surgeons who implant hips and their representative professional bodies the British Orthopaedic Association (BOA) and the British Hip Society (BHS). In addition, the MHRA appointed a Metal-on-Metal Expert Advisory Group (EAG) in 2008 (MHRA 2010b), comprised of representatives from two manufacturers, clinicians delegated by the BOA and the BHS, and MHRA staff.

#### The manufacturer

DePuy Orthopaedics was aware that data on the ASR™ in the Australian Joint Registry from 2007 onwards, and the NJR of England and Wales from 2009, showed higher revision rates than equivalent MoM hip products. The company’s attention was also explicitly drawn to these high revision rates, and therefore to the real risk to patient health, on several occasions in conference presentations, private correspondence and meetings (Cohen 2011). DePuy’s first response was to displace responsibility from any problem with the implant by advising surgeons to alter their implantation technique (ibid.). Manufacturers have greater access to data on their products than any other stakeholder does, in addition to extensive access to NJR data; they have internal data on device design and feedback from surgeons on performance, meaning they should have a fuller picture of how an implant is performing. It is not known how DePuy used this privileged access to data above and beyond referencing implantation technique. However, the company cited unpublished NJR data as one of the reasons for their worldwide withdrawal of the ASR™ in August 2010 (DePuy Orthopaedics Inc. 2010). Beyond external inputs to the manufacturer there were a number of internal studies commissioned by DePuy between 2005 and 2011 that warned of the engineering deficits of the ASR™ implants (Cohen 2012b). It is a source of conjecture among patients globally as to how the ‘ASR story’ might have unfolded if DePuy had acted on known problems and withdrawn the implant sooner than it did.

It should be recognised that DePuy have never accepted that there has been a defect with the ASR™ hip, and this has been shown in the evidence they have offered in USA courts. For example, it was reported that, at a trial in March 2013, ‘J&J attorney Richard Sarver said the device was not defective’ (Bloomberg 2013a). Evidence presented in the same court brings into question whether DePuy Orthopaedics took seriously, and was transparent about, data which showed the implant could be harmful to patients (Meier 2013). In a major class action in the USA, at the end of 2013, Johnson and Johnson offered a settlement to around 8,000 litigants, estimated at US$2.47 billion (Bloomberg 2013b). In Australia, a legal class action was due to commence in early June 2014 but was postponed until March 2015 when DePuy submitted 23,000 pages of additional documentation in what an ASR™ patient described as ‘delay by avalanche’ (The Australian 2014). As such, DePuy Orthopaedics (Johnson & Johnson) have fought and continue to fight expensive and long-running legal battles to defend the ASR™.

In addition, government enquiries have questioned DePuy’s approach to dealing with problems associated with the ASR™. The Australian Senate Community Affairs References Committee’s enquiry into ‘The regulatory standards for the approval of medical devices in Australia’ (2011) voiced their concern:

The committee is deeply disturbed by what appears to be tardiness on the part of JJM [Johnson & Johnson Medical, the parent company of DePuy] to act on known problems with these devices. Many people could have avoided considerable pain, suffering and diminished quality of life if the company had acted in a responsible manner to known problems with these devices. In failing to respond to the committee’s requests for information on this matter, JJM have only served to confirm the committee’s views. (page 74)

In the UK, the House of Commons Science and Technology Committee noted during their enquiry into the 'Regulation of medical implants in the EU and UK' (2012: 6) that they were ‘very disappointed that we were not able to take oral evidence directly from manufacturers’ about hip implant issues. This evidence suggests that, although medical device and pharmaceutical companies routinely claim that helping patients is at the centre of their operations (Johnson & Johnson Medical Pty Ltd 2011: 4), a profit-led business model is in place and is prioritised over protecting patients (compare with Barbui et al. 2011; Gøtzsche and Jørgensen 2011; Rosch 2012).

#### The regulator

The MHRA is an executive agency of the UK’s Department of Health whose task is to ‘safeguard public health through our primary role in ensuring that the products we regulate meet required standards, that they work and are acceptably safe’ (MHRA 2013). In the case of the ASR™ hip it is therefore reasonable to ask why the MHRA did not take precautions to safeguard the public’s health given mounting evidence about implant failure.

Its MoM Expert Advisory Group (EAG) had been reporting more generally on MoM hip replacements since 2008 and, it was claimed, met ‘regularly to evaluate new scientific advice and observations from clinicians’ (MHRA 2012b). The EAG has only two published outputs – advice on patient management and a summary report released in October 2010, which looked at a range of MoM designs, but omitted the DePuy ASR™ implants (MHRA 2010b). As the ASR™ implants are a MoM design, one would expect a discussion on emerging data on ASR™ failures to have been part of the Group’s work. Furthermore, if the Group met regularly, more information on their work should be available, for example the minutes of meetings whereby the Group’s discussions, analyses and progress might be determined. A request for information submitted to the MHRA by the authors of this paper asking for access to relevant minutes, has not been answered at the time of writing, although minutes from a predecessor EAG are publicly available. If these records exist and the EAG was regularly briefing MHRA staff, why are such deliberations not made public? This is particularly significant when taking into account that key stakeholders such as the BOA cite delegating experts to the EAG as one of their core contributions in the ASR™ case:

The information on failures of Metal on Metal implants accumulated slowly – as is always the case – but came to a point when, based on Joint Registry data in Australia and the UK, one implant in particular – De Puy’s ASR system – was identified as performing significantly less well than others used to replace painful arthritic hip joints. The BOA brought this to the attention of the MHRA, helped provide senior and expert clinical input into the Expert Advisory Group on Metal on Metal Implants, and addressed the question of how to monitor implants introduced by forming the Orthopaedic Devices Evaluation Panel. (BOA British Orthopaedic Association 2014)

In its report on medical implant regulation, the House of Commons’ Science and Technology Committee raised similar concerns about secrecy around the work of another of the MHRA’s internal deliberation bodies, the Committee on the Safety of Devices (CSD)^b^ (House of Commons Science and Technology Committee 2012), which may indicate an institutional attitude of hesitancy in sharing on-going deliberations on medical implants. It seems professionally remiss not to be making use of the combined expertise of a group whose task is so closely linked to the unfolding of ‘one of the biggest disasters in orthopaedic history’ (Cohen 2011). The EAG might have been able to operate as the key protector of patient safety, helping to minimise the effects of the unfolding ASR™ failure.

The MHRA has a statutory leadership role in engaging with medical implant failures but there is little evidence of it having provided leadership in ensuring patient well-being related to the ASR™ hip replacement, which might have been pivotal in safeguarding the health of those patients about to be implanted with the ASR™ in the UK and perhaps in other countries, too. The MHRA’s commitment to leading on ensuring the safety of medical implants has been shown to be weak and their inaction has been criticised elsewhere (Horton 2012). Yet the agency continues to see its actions as not just blameless but commendable. The MHRA argues that it ‘was the first regulatory agency in the world to issue advice to clinicians about MoM hip implants in April 2010’ (MHRA 2012c) and that it acted with precaution:

As a *precautionary* measure, we have today issued updated patient management and monitoring advice to surgeons and doctors … because this particular type of hip replacement has a small risk of causing complications in patients. (MHRA 2012b, our emphasis)

If the MHRA had been committed to precautionary governance of the implant with patient safety in mind, it could have called for a moratorium on ASR™ implantation based on data publicly released in the 2009 NJR report. Even such a date is relatively late given that there had been concerns articulated in the Australian registry in 2007 and in journal publications from 2008 (Langton et al. 2008; Joyce et al. 2009). It was these and subsequent journal publications which not only raised concerns over the performance of the ASR™ but explained why the ASR™ hip was performing so poorly, essentially due to a design defect which led to high wear of the MoM implant (Langton et al. 2008; Joyce et al. 2009; Langton et al. 2011). Therefore, a definitive cause of poor performance by the ASR™ was proposed and was available to counter any arguments regarding technical uncertainty.

#### The NJR

Set up in 2002, the NJR ‘collects information on joint replacement surgery and monitors the performance of joint replacement implants’ (NJR 2012: 15). Nearly a quarter – 23% – of its Steering Committee representation is from industry. The registry publishes annual reports on the performance of implant surgeries and claims to be able to identify poorly performing implants quickly. For example, the Chairman’s Introduction to the 2009 report states:

As a result of outlier analysis by the NJR, the Medicines and Healthcare products Regulatory Agency (MHRA) issued a device alert for an implant which has subsequently been withdrawn by the manufacturer. We can now quickly inform hospitals of potential problems, which significantly reduces the period between identification of an issue and patient review. I believe we have demonstrated that the Register is a tool for excellence and that it will enable continuing improvement in best practice and patient care. (NJR 2009: 8)

The NJR therefore make an explicit link between their identification of high revision rates and subsequent action taken by the MHRA to remove poorly performing implants from the market (although the implant they are referring to above is not named). The same 2009 report names the ASR™ as the hip resurfacing implant with the highest revision rate (7.5% failure at three years, more than twice the NICE recommendation) and yet the ASR™ was only removed from the market August 2010 *by the manufacturer.* The NJR’s claim to be able to identify and remove poor implants from the market efficiently and quickly, does not therefore appear to be borne out in the case of the ASR™. This is confirmed in an analysis by authors who sit on the NJR Steering Committee:

The NJR did not confidently determine that there was a potential problem with the ASR™ implant until the 2008 data were analyzed. The NJR reported the ASR™ implant to the MHRA in April 2010, at which time an MHRA device alert was issued. (Tucker et al. 2011)

This analysis was effectively presented too late for those patients who were implanted with the ASR™ hip throughout 2009 and 2010, as well as those implanted earlier and reporting problems to their GPs and orthopaedic surgeons, and brings into question the NJR’s ability, at that time, to be a tool for rapid identification of implants with high revision rates.

#### Professional bodies and orthopaedic surgeons

Surgeons tend to draw on guidance provided by their professional bodies, in this case the British Orthopaedic Association (BOA) and its specialist offshoot the British Hip Society (BHS). Prior to ASR™ withdrawal in 2010, neither body published formal guidance or recommendations on the accumulating failure data. Instead, the BOA argues that it engaged with the MHRA, in raising the regulator’s awareness of the ASR™ ‘performing significantly less well than others’ (BOA [British Orthopaedic Association] 2014). In addition, it funded important research investigating the poor clinical performance of the ASR™. The BHS now recognises it could have acted differently, stating that the professional bodies need to ‘be proactive if similar problems arise again’ (Bannister 2012). It is clear that surgeons responded individually to data about ASR™ failure which emerged during professional conferences and in surgical journals:

Data critical of the performance of the ASR were presented at numerous scientific meetings in 2008 and onward to the time of this writing, particularly at meetings of the British Hip Society (BHS) and the British Orthopaedic Association (BOA). This factor, rather than the MHRA alert in July 2010, almost certainly led to the subsequent drop in sales of that implant. (Tucker et al. 2011: 40)

Orthopaedic surgeons opted to implant alternative hips to the ASR™ and it is probable that the collective effect of these individual decisions impacted ASR™ sales figures, leading to declining sales and the manufacturer’s decision to withdraw the implant: ‘DePuy decided in 2009 that it would be discontinuing the ASR™ System as a result of declining demand’ (DePuy Orthopaedics Inc. 2010; see also: Bloomberg 2013c).

Some orthopaedic surgeons have recently introduced an initiative to allow a step-wise, more controlled introduction for new implants in parallel to existing governance, called Beyond Compliance (http://www.beyondcompliance.org.uk). While this step is to be lauded, the website lists only eight orthopaedic products (as of 23 October 2014), compared with 256 brands of hip implants (acetabular cups and femoral stems) alone that were used in the UK in 2013 (NJR 2014: 67)^c^. It remains to be seen if this initiative will find wider uptake in the future.

## Governance failure as precautionary uncertainty

Whilst each stakeholder in hindsight seeks to explain or defend their actions, the ASR™ hip implant history speaks of a failure of governance in protecting patients. Despite the MHRA retrospectively citing precautionary action in regard to patient management the difficulty of ascertaining what constitutes appropriate precautionary action remains (cf. Ahteensuu 2004). In practical terms, this means decision makers have to choose which group or aspect of the system they prioritise for precautionary actions. Recent discussion about precautionary approaches reveals wide gaps in the perception of what precaution means for the governance of innovative science and technology (Bell 2013; Brown 2013; Fuller 2013; Stilgoe 2013; Stirling 2013). One understanding of precaution recurrent in ethical deliberations is responsibility towards persons and humankind. Another understanding, prevalent in European policy discourse of recent decades, has shifted responsibility towards ensuring specific social relationships, such as a free market, a stable and competitive economy, and civic subsidiarity. These two understandings of precaution may not always compete, but in the case of medical implants they have arguably rendered technological decision-making contentious. In the case of the ASR™ hip, the data that was emerging should have provided a basis for developing a precautionary assessment, particularly from the regulator and the manufacturer. The uncertainty around this data which the MHRA and other stakeholders use as justification for not calling for a moratorium on implantation, should actually have been deemed sufficient to employ precaution in order to protect patients over macroeconomic or other systemic considerations. For example, data showing the ASR™ had high revision rates compared to other resurfacing hips was publicly available from 2007 onwards, yet at no time was it considered definitive enough to cease implantation – this only occurred with withdrawal of the implant by the manufacturer. Such uncertainty can lead to considerable impact on those people who are most vulnerable, i.e. patients, when the object of precaution – the stakeholder seen as meriting protection – is not the vulnerable person or group but the enacted system incorporating the problem.

How can the balancing of responsibilities be understood in the case of the ASR™? First, the perspective of the regulator is shaped by its routine interactions. The medical implant system is part of a larger healthcare system and consists of diverse social relationships between regulatory and other governance bodies, commercial organisations, practitioner stakeholders such as surgeons, and patients. These interactions contribute to the perpetuation of the governance system through informing, deliberating and decision-making. Whilst patient safety has been written into the regulator’s mission, there appears to have been little responsiveness to concerns and problems of patients implanted with ASR™ hips, by MHRA decision-makers. If experiential data from patients had been assessed alongside quantitative data from the NJR, this should have made a difference to the regulator’s assessment of which group to prioritise for precautionary action. Second, the MHRA places their responsibility for medical implant safety in the context of upholding the viability and reliability of the governance system, by keeping regulatory decisions confidential as suggested by their response to the Freedom of Information request by the BMJ (Cohen 2011), and by including manufacturers, healthcare professionals and the public in the post-market monitoring of implant safety (e.g. MHRA 2012e: 10). It is our contention that the MHRA should refocus their attention on patients and prioritise their relationship with them over its myriad others. Third, hip replacements have received much praise over the last decades, with reliability lauded and improvement to patients’ lives widely accepted in society. Confidence in the hip replacement is emblematic of the success of the UK healthcare system and its governance yet when a product such as the ASR™ produces considerable adverse effects, confidence in medical implants is damaged. A quick decision on halting implantation of the ASR™ and efficient management of those affected could have preserved confidence in hip implants and the implant governance system in the UK.

Different perspectives, as different realities, shape expectations of what constitutes adequate precautionary action. The MHRA presents itself as a champion of patients, but operates as a champion of what can be described as the status quo between statistically safe healthcare provision and economic growth through medical devices and drugs innovation (compare with Clark 2013). Different understandings of precautionary action aside, the case of the ASR™ shows that patient safety should have priority over other concerns as medical implants have very material, direct and long-lasting impact on patients and their families.

### The ASR™ as a ‘public experiment’

Social studies of science and technology have suggested that contemporary development and deployment of new technologies increasingly takes place in wider social spaces rather than in the protected space of the scientific laboratory. In such a ‘technological society’ (Barry 2001) the experimental space has extended to include the market and consumers: society itself operates as a laboratory (Krohn and Weyer 1994; Nordmann 2009). One corollary of this development is that technoscientific innovation can easily outpace its governance. In the experimental space of society such innovations emerge in interplay with a wide range of social, institutional and health-related changes and developments. They tend to be applied without adequate capacity to develop understanding of the potential risks and impacts on health, behaviour, and practice. What amounts to the continuation of testing in the market makes understanding and considering precautionary approaches crucial, so that in cases of failure, existing and potential users of that technology can be protected, fully informed, and not retained as further experimental subjects. One ASR™ patient perspective brings this to life:

It is difficult as a victim to come to terms with the fact that, if I had received one of the many, many proven hips that are available, I would be enjoying life without the pain and suffering that will follow me for the rest of my life. It would have been that easy. No second five–hour operation, no bone grafts, no splitting of my femur open and putting it together with clamps and wire-caging, no pneumonia, no renal failure, no second stay in intensive care, no pain, no pain–killing drugs, no stress for my wife and family and no loss of what will amount to a minimum of $80,000 to myself and the community–all because someone wanted, for whatever inducement, to use me in a medical trial, and I am one of the lucky ones. They value the quality of our lives too cheaply. (Australian Senate Community Affairs References Committee 2011: 54)

A lack of patient-centred precaution has been traced in the case of the ASR™ and other MoM hip implants resulting in a public experiment.

Despite the fact that these risks have been known and well documented for decades, patients have been kept in the dark about their participation in what has effectively been a *large uncontrolled experiment*. (Cohen 2012b: 1 – our emphasis)

In the case of the ASR™ hip, the lack of clinical trials and transparency around in-vitro testing of hip replacements pre-market means that most data is collected during market-wide use in patients. While institutions such as the MHRA and the NJR exist to monitor post market use and respond to problems, the ASR™ hip case shows that the governance parameters of this experiment were too unclear to ensure patient safety. What renders science and technology governance more complicated is the uncertainty inherent to these fields. For example, the NJR note that they may have drawn attention to ASR™ failures sooner had their method of outlier analysis been more precise (NJR 2009: 12). This suggests that even control mechanisms themselves are processes that require on-going refinement and can help to anticipate and respond only to a certain degree.

The uncertainty in deciding on action that is most appropriate for responding to an emerging problem is aggravated by the normativity of public experiments in which responsiveness to data is dependent on very visible outliers and that complications are visible in the majority of patients (MHRA 2012a, 2012c, 2012d). The case of the ASR™ hip shows that the notion of society as a public laboratory, or experiment, becomes problematic when opaque regulatory guidelines and monitoring processes cloud awareness of problems reported by patients and surgeons by apparently ignoring some avenues of information and prioritising others, and by not acknowledging that both the implant and its governance are subject to uncertainty. This presents a state that may require precautionary responsiveness in favour of those that are most affected by failure of implants, the patients.

### Policy-commerce alignment

In the technological society the variability of governance parameters, and as such disagreement as to what precautionary action encompasses, is further affected by the alignment of policy with commercial interests where the notion of precaution can be imagined as stifling innovation or as being unscientific. The recent inauguration of an Innovation Office by the MHRA to support manufacturers in bringing new products to the market is an indicator of the agency’s priorities and commitment to commercial interests (Clark 2013).

It has been argued that commercial companies increasingly contribute to the development of regulatory and testing regimes, to significantly influence the aim and scope of testing systems that are part of technological regulation (Abraham and Ballinger 2012; Lave et al. 2010). Their capacity to take such an influential role is contextualised by a dominance of economic concerns that are expressed in free-market, neo-liberal thinking: science and technology as well as innovation policy stakeholders have adopted a commercial prerogative, which in turn impacts on the governance of science and technology research, development and deployment (RD&D) in society. What is seen is the tendency to protect commercial interests through policy and regulation, and an effort to retain the status quo of confidence in existing regulation that allows as much space for commercial RD&D as possible (cf. Di Mario et al. 2011).

Similarly, in cases of demonstrable and wide-reaching failure, the commercial prerogative impacts on the ability to govern that failure, resulting in ‘civil dislocation’: the mismatch between the functions that government institutions are supposed to perform and their actual practice (Jasanoff 1997). In the ASR™ hip case it is possible to talk of ‘regulatory rigor mortis’ (Van Zwanenberg and Millstone 2005: 50) as such a civil dislocation. No single stakeholder has resolutely responded to data on high revision rates with a patient-centred precautionary approach that could have put patient safety before commercial or other concerns. This was aggravated by a lack of de facto independence of stakeholders (Busuioc et al. 2011; Makhashvili and Stephenson 2013), who are part of a network of actors in which contributions and thus responsibility are shared. When regulatory bodies such as the MHRA have to balance patient well-being against the economic interests of industry, then a precautionary decision in favour of patient safety may be seen as having negative commercial impact. However, it can be argued that early intervention in the ASR™ case would have reduced economic and confidence costs in the long run.

## Conclusion

The precarious balance arising from precautionary uncertainty is the tip of the iceberg of a systemic failure. The inability of regulators to respond robustly to emergent problems and their consequences in the ASR™ hip case is evidence of a medical implant governance system that is not capable of protecting patient safety despite this being articulated as its raison d’être. Instead, the stated independent position of the regulator – independent from other actors – becomes visible for what it actually is: a tenuous network of competing influences, interests, roles and responsibilities in which the safety of a medical implant is constructed through the interpretation of its performance according to underlying commercial interests. Post market surveillance is a significant aspect of manufacturer governance of medical implants^d^. They operate thus because other interested actors within the system, including the regulator (see, e.g., BBC Newsnight 2012; Woods 2012), subscribe to the mechanism of the societal laboratory^e^ and because pre-market authorisation structures, such as substantial equivalence, are weak (Cohen 2012a). In the UK the implant governance system failed to learn thoroughly from the 3M™ Capital™ Hip failure, despite claims by the MHRA in 2012 of having responded to emerging ASR™ data with patient safety in mind (MHRA 2012b). There is little evidence as to the regulator’s internal negotiation of the situation, which is staggering, given their role as a public body and the effect on patients’ lives. In the response to the emergent and early indication of implant failure data we can see a network of various stakeholders whose actions may be inhibited by: the potential of the manufacturer’s legal response (Channel 4 2011); the regulator’s inherent alignment with commercial imperatives; and institutional ambiguity about risks and remits.

We agree with Abraham and Ballinger (2012) that governance and commercial interests align in the health care context, and that this works to the detriment of patient safety. We suggest that this is an inherent and entrenched aspect of medical implant governance in the UK and the EU biased by the commercial prerogative of an economic European Community in the context of global economic competitiveness. The very recent discussion of moving drugs and medical devices to the enterprise portfolio of the EU, rather than health, only serves as additional evidence (European Public Health Alliance 2014).

Key stakeholders in the ASR™ story took too long to respond to emergent intelligence and evidence of increased revision rates of the ASR™ hip design. The response by the MHRA and the manufacturer, favouring commercial interests and confidence in the existing status quo has played out to the detriment of patients and the notion of health-*care* in the UK.

### Endnotes

^a^ NICE guidance was updated in February 2014, stating that ‘Only an artificial hip or hip resurfacing with a replacement rate of less than 1 in 20 at 10 years should be used’: http://www.nice.org.uk/guidance/ta304.

^b^ Incidentally, the CSD commissioned the EAG on ‘Biological effects of metal wear debris generated from hip implants: genotoxicity’ in 2006, in response to a report by the Department of Health’s independent expert advisory Committee on Mutagenicity of Chemicals in Food, Consumer Products and the Environment (COM). COM’s report found ‘good evidence for an association between [metal-on-metal and metal-on-polyethylene] hip replacements and increased genotoxicity in patients’ (EAG 2006: 2). The EAG on genotoxicity was set up ‘with the remit of translating the conclusions reached by the COM into advice for clinicians and patients’ (ibid.). In its last set of minutes this EAG noted problems with soft tissue reactions from metal debris (EAG 2010), leading the MHRA to set up a new EAG dedicated to assessing the significance of soft tissue necrosis associated with MoM hip replacements. In this paper we look at this last EAG, which met between January 2008 and March 2010.

^c^ Updated information on prostheses used in hip, knee, ankle, elbow and shoulder replacements, as registered by the NJR, can be found at this web address: http://www.njrreports.org.uk/implants. The latest available data stems from 2013.

^d^ All devices require the ‘CE’ mark before entering the European market (The Council of the European Communities 1993). With the submission for and the award of the CE mark the device’s manufacturer (including those involved in device packaging, labelling and distributing) accepts sole responsibility for the device and its functioning (The European Parliament, and The Council of the European Union 2008).

^e^ For example, the head of the MHRA claims that hip joints cannot be tested outside the human body, despite machines to allow laboratory testing being first developed in 1966.

## Abbreviations

ASR™: Articular Surface Replacement (trademarked hip design)
BHR™: Birmingham Hip Resurfacing (trademarked hip design)
BHS: British Hip Society
BOA: British Orthopaedic Association
CE: Not expanded, taken to mean ‘Conformité Européenne’ (conformity mark in the European Economy Area)
COM: Committee on Mutagenicity of Chemicals in Food, Consumer Products and the Environment (independent advisory body to the Department of Health)
CSD: Committee on the Safety of Devices (internal advisory body to the MHRA)
EAG: Expert Advisory Group
EU: European Union
FDA: Food and Drug Administration, USA
MDA: Medical Device Alert
MHRA: Medicines and Healthcare products Regulatory Agency, UK
MoM: Metal-on-Metal (hips)
NICE: National Institute for Clinical Excellence
NJR: National Joint Registry of England, Wales and Northern Ireland
RD&D: Research, Development and Deployment
UK: United Kingdom
